# Construction of Optimal Two-Dimensional Optical Orthogonal Codes with at Most One Pulse per Wavelength

**DOI:** 10.3390/e26090741

**Published:** 2024-08-30

**Authors:** Minfeng Shao, Xianhua Niu

**Affiliations:** School of Computer and Software Engineering, Xihua University, Chengdu 610097, China; rurustef1212@gmail.com

**Keywords:** two-dimensional optical orthogonal code (2D OOC), optical orthogonal code (OOC), optical code division multiple access network

## Abstract

Two-dimensional optical orthogonal codes have important applications in optical code division multiple access networks. In this paper, a generic construction of two-dimensional optical orthogonal codes with at most one pulse per wavelength (AM-OPPW 2D OOCs) is proposed. As a result, some optimal AM-OPPW 2D OOCs with new parameters can be yielded. The new AM-OPPW 2D OOC may support more subscribers and heavier asynchronous traffic compared with known constructions.

## 1. Introduction

With the advantage of combining the large transmission bandwidth of fiber-optic media and the flexibility of code division multiple access (CDMA) techniques, the optical code division access system (OCDMA) has been extensively studied since the 1980s. In this system, unipolar {0,1} optical orthogonal codes (OOCs [1]) are employed as spreading codes. However, this multiple-access scheme has a drawback in that the effect of multiple-access interference (MAI) cannot be completely eliminated as in directly spreading CDMA systems. Thus, one of the key points for the OCDMA system is to design OOCs with low cross-correlation and off-peak autocorrelation. In the meantime, to enlarge the discrimination between the correct codeword and interfering codewords, we also need a large peak autocorrelation value, i.e.,  the weight of the OOCs. Finally, since the number of users in the system is less than or equal to the code size of OOCs, it is beneficial to design OOCs with large code sizes. However, as the volume of codewords or the weight of the code increases rapidly, the code length increases rapidly. Thus, optimal solutions, i.e., optimal OOCs, were proposed with respect to the tradeoff of those parameters (see, e.g., [2,3,4,5,6,7,8,9,10]).

In the meantime, two-dimensional optical orthogonal codes (2D OOCs) that spread in both time and wavelength were introduced for the OCDMA system to overcome this drawback. Similarly, to minimize multiple-access interference, we have to minimize the cross-correlation and off-peak autocorrelation of 2D OOCs; to support a large number of users, we need a large set of 2D OOCs. Moreover, to simplify the practical implementations, restrictions, such as at most one pulse per wavelength (AM-OPPW) and at most one pulse per time slot (AM-OPPTS), are often imposed on 2D OOCs [11]. However, these parameters are not independent of each other. They suffer some theoretic bounds, for instance, the Johnson bound [4] and the bound for 2D OOCs with the AM-OPPW restriction [11]. So far, various works have addressed optimal 2D OOCs with respect to these bounds [11,12,13,14,15,16,17,18,19,20,21,22,23,24,25,26,27].

The main idea of this paper is to generate new optimal AM-OPPW 2D OOCs based on known OOCs and 2D OOCs. In [16,17,19], OOCs were used to construct 2D OOCs by spreading them in the time domain, i.e., the OOCs form rows of 2D OOCs. In our construction, the OOCs are utilized to determine which rows of 2D OOCs are not all-zero vectors. In this way, new AM-OPPW 2D OOCs can be yielded with large sizes, some of which are optimal with respect to the theoretic bound proposed in [11]. Further, we also analyze the performances of the new 2D OOCs under the chip-synchronous and chip-asynchronous assumptions, respectively.

The remainder of this paper is organized as follows. Section 2 reviews some necessary preliminaries. Section 3 introduces the new construction of AM-OPPW 2D OOCs. Section 4 conducts the performances of the new AM-OPPW 2D OOCs under the chip-synchronous and chip-asynchronous assumptions, respectively. Section 5 concludes this paper.

## 2. Preliminaries

Let Λ, *T*, *w*, and λ be positive integers, and ${\langle a\rangle }_{b}$ be the least non-negative residue of *a* modulo *b* for positive integers *a* and *b*. A (Λ×T,w,λ) *two-dimensional optical orthogonal code* (2D OOC) C is a family of {0,1} arrays of order Λ×T with constant weight *w* satisfying the following two properties:

(1) The autocorrelation property
  (1) $$\begin{array}{cc} R_{X,X}\left(\tau \right)= & \sum_{k=0}^{\Lambda -1}\sum_{t=0}^{T-1}X_{k,t}X_{k,{\langle t+\tau \rangle }_{T}}\leq \lambda ,\;0<\tau \leq T-1, \end{array}$$
(2) The cross-correlation property
  (2) $$\begin{array}{cc} R_{X,Y}\left(\tau \right)= & \sum_{k=0}^{\Lambda -1}\sum_{t=0}^{T-1}X_{k,t}Y_{k,{\langle t+\tau \rangle }_{T}}\leq \lambda ,\;0\leq \tau \leq T-1, \end{array}$$

where $X={\left(X_{k,t}\right)}_{0\leq k<\Lambda ,0\leq t<T}\in C$, $Y={\left(Y_{k,t}\right)}_{0\leq k<\Lambda ,0\leq t<T}\in C$ and X≠Y.

If λ is the smallest integer such that (1) and (2) hold, then we say that λ is the *maximum collision parameter* (MCP) of C. If Λ=1, then C is exactly an *optical orthogonal code* (OOC) [1].

The following restrictions on the placement of pulse within an array are often proposed for 2D OOCs to simplify the practical implementations [11]:

- Arrays with one pulse per wavelength (OPPW): For any array *X* in C, the element 1 appears exactly once in each row of *X*.
- Arrays with at most one pulse per wavelength (AM-OPPW): For any array *X* in C, the element 1 appears at most once in each row of *X*.
- Arrays with one pulse per time slot (OPPTS): For any array *X* in C, the element 1 appears exactly once in each column of *X*.
- Arrays with at most one pulse per time slot (AM-OPPTS): For any array *X* in C, the element 1 appears at most once in each column of *X*.

Obviously, OPPW (OPPTS resp.) is a special case of AM-OPPW (AM-OPPTS resp.).

In the following, we briefly review the theoretic bounds on the code size of OOCs and 2D OOCs with AM-OPPW.

**Lemma** **1** ([4])**.** *The maximum possible size Φ(1×T,w,λ) of an OOC with parameters (1×T,w,λ) is bounded by*

$$\Phi \left(1\times T,w,\lambda \right)\leq \left\lfloor \frac{1}{w}\left\lfloor \frac{T-1}{w-1}\left\lfloor \frac{T-2}{w-2}\ldots \left\lfloor \frac{T-\lambda }{w-\lambda }\right\rfloor \ldots \right\rfloor \right\rfloor \right\rfloor .$$

**Lemma** **2** ([11])**.** *The maximum possible size Φ(Λ×T,w,λ) of a (Λ×T,w,λ) 2D OOC with AM-OPPW is bounded by*

$$\begin{array}{cc} \Phi (\Lambda \times T,w,\lambda )\leq & \left\lfloor \frac{\Lambda }{w}\left\lfloor \frac{T(\Lambda -1)}{w-1}\left\lfloor \frac{T(\Lambda -2)}{w-2}\ldots \left\lfloor \frac{T(\Lambda -\lambda )}{w-\lambda }\right\rfloor \ldots \right\rfloor \right\rfloor \right\rfloor . \end{array}$$

*In particular, for the 2D OOC with OPPW,*

$$\Phi \left(\Lambda \times T,w,\lambda \right)\leq T^{\lambda }.$$

An OOC (a 2D OOC with AM-OPPW resp.) is called *optimal* if the number of codewords achieves the theoretic bound in Lemma 1 (Lemma 2 resp.).

## 3. Optimal AM-OPPW 2D OOCs via Known AM-OPPW 2D OOCs and OOCs

In this section we introduce a general construction of AM-OPPW 2D OOCs based on known OOCs and AM-OPPW 2D OOCs.

Let $C=\{C^{0},C^{1},\ldots ,C^{M-1}\}$ be an AM-OPPW 2D OOC with parameters (Λ×T, $\Lambda ,\lambda_{1})$. For 0≤i<M, $C^{i}$ is defined by

$$\begin{array}{c} C^{i}={\left({C_{0}^{i}}^{⊤},{C_{1}^{i}}^{⊤},\ldots ,{C_{\Lambda -1}^{i}}^{⊤}\right)}^{⊤}=\left(\begin{array}{ccc} c_{0,0}^{i}, & c_{0,1}^{i}, & \ldots ,c_{0,T-1}^{i}\\ c_{1,0}^{i}, & c_{1,1}^{i}, & \ldots ,c_{1,T-1}^{i}\\ \vdots & \vdots & \vdots \\ c_{\Lambda -1,0}^{i}, & c_{\Lambda -1,1}^{i}, & \ldots ,c_{\Lambda -1,T-1}^{i} \end{array}\right), \end{array}$$

where ⊤ is the transpose operation, and the *T*-dimensional vector $C_{j}^{i}=\left(c_{j,0}^{i},c_{j,1}^{i},\ldots ,c_{j,T-1}^{i}\right)$ is the (j+1)-th row of the array $C^{i}$, 0≤j<Λ. Let $S=\{S^{0},\ldots ,S^{N-1}\}$ be an OOC with parameters $\left(n,\Lambda ,\lambda_{2}\right)$, where $S^{r}=\left(s_{0}^{r},\ldots ,s_{n-1}^{r}\right)$ for 0≤r<N.

With the above preparation, we can construct an AM-OPPW 2D OOC $X=\{X^{\left(C^{i},S^{r},j\right)}\;|$$\;C^{i}\in C,\;S^{r}\in S,\;0\leq i<M,\;0\leq r<N,0\leq j<n\}$ as follows.

**Construction A:** For each three-tuple (i,r,j), 0≤i<M,0≤r<N,0≤j<n, run the following Algorithm 1 to generate a new n×T array $X^{\left(C^{i},S^{r},j\right)}$:
**Algorithm 1** Generate the new array**Input:** $C^{i}$, $S^{r}$, *j*. **Initiate:** τ=0, t=0; **while** 
0≤k<n 
**do**  **if** $s_{k+j}^{r}=1$ **then**   $X_{k}^{\left(C^{i},S^{r},j\right)}=C_{\tau }^{i}$;   τ=τ+1;  **else**   $X_{k}^{\left(C^{i},S^{r},j\right)}=0;$ // **0** is the all-zero *T*-dimensional vector  **end if**  k=k+1; **end while**
 **return** 
$X^{\left(C^{i},S^{r},j\right)}={\left(X_{0}^{\left(S^{r},C^{i},j\right)},X_{1}^{\left(S^{r},C^{i},j\right)},\ldots ,X_{n-1}^{\left(S^{r},C^{i},j\right)}\right)}^{⊤}.$

**Theorem** **1.** *The 2D OOC X generated by Construction A is an AM-OPPW 2D OOC with parameters (n×T,w,λ), code size nNM, and $\lambda \leq \max\{\lambda_{1},\lambda_{2}\}$.*

**Proof.**  We first show that the MCP of X is less than or equal to $\max\{\lambda_{1},\lambda_{2}\}$. By (1) and (2), it is sufficient to investigate

$$\begin{array}{c} R_{X^{\left(C^{i_{1}},S^{r_{1}},j_{1}\right)},X^{\left(C^{i_{2}},S^{r_{2}},j_{2}\right)}}\left(\tau \right)=\sum_{k=0}^{\Lambda -1}\sum_{t=0}^{T-1}X_{k,t}^{\left(C^{i_{1}},S^{r_{1}},j_{1}\right)}X_{k,{\langle t+\tau \rangle }_{T}}^{\left(C^{i_{2}},S^{r_{2}},j_{2}\right)}, \end{array}$$

which is divided into two cases according to the values of *i*, *r*, and *j*.

Case I: $\left(r_{1},j_{1}\right)\neq \left(r_{2},j_{2}\right)$. By Algorithm 1, the rows $X_{k}^{\left(C^{i_{1}},S^{r_{1}},j_{1}\right)}\neq 0$ and $X_{k}^{\left(C^{i_{2}},S^{r_{2}},j_{2}\right)}\neq 0$ if and only if $s_{k+j_{1}}^{r_{1}}=1$ and $s_{k+j_{2}}^{r_{2}}=1$, where 0≤k<n. Note that both $X_{k}^{\left(C^{i_{1}},S^{r_{1}},j_{1}\right)}\neq 0$ and $X_{k}^{\left(C^{i_{2}},S^{r_{2}},j_{2}\right)}\neq 0$ contain at most one element 1. Thus, the cross-correlation value $R_{X^{\left(C^{i_{1}},S^{r_{1}},j_{1}\right)},X^{\left(C^{i_{2}},S^{r_{2}},j_{2}\right)}}\left(\tau \right)$ is less than or equal to the correlation of $S^{r_{1}}$ and $S^{r_{2}}$ at time shift $j_{2}-j_{1}$, i.e., ≤$\lambda_{2}$.

Case II: $\left(r_{1},j_{1}\right)=\left(r_{2},j_{2}\right)$. By Algorithm 1, $X_{k}^{\left(C^{i_{1}},S^{r_{1}},j_{1}\right)}\neq 0$ and $X_{k}^{\left(C^{i_{2}},S^{r_{1}},j_{1}\right)}\neq 0$ are rows of $C^{i_{1}}$ and $C^{i_{2}}$, respectively. Hence, the correlation value $R_{X^{\left(C^{i_{1}},S^{r_{1}},j_{1}\right)},X^{\left(C^{i_{2}},S^{r_{1}},j_{1}\right)}}\left(\tau \right)$ is less than or equal to the correlation of $C^{i_{1}}$ and $C^{i_{2}}$ at time shift τ. Then, the nontrivial correlation value $R_{X^{\left(C^{i_{1}},S^{r_{1}},j_{1}\right)},X^{\left(C^{i_{1}},S^{r_{1}},j_{1}\right)}}\left(\tau \right)$, i.e., $i_{1}\neq i_{2}$ or ($i_{1}=i_{2}$ and τ≠0(modT)), is less than or equal to $\lambda_{1}$.

In addition, it is easy to check that |X|=nNM. Therefore, the AM-OPPW 2D OOC X has parameters (n×T,Λ,λ) and size nNM, where $\lambda \leq \max\{\lambda_{1},\lambda_{2}\}.$ □

In what follows, we present some results obtained by Construction A for specific cases of λ=1,2. Firstly, for λ=1, we have the following result:

**Corollary** **1.** *If C is an optimal OPPW 2D OOC with parameters (Λ×T,Λ,1) and S is an optimal OOC with parameters (n,Λ,1), then the AM-OPPW 2D OOC X generated by Construction A with parameters (n×T,Λ,1) is optimal for T≥Λ and Λ(Λ−1)∣(n−1).*

**Proof.**  Because of the optimality of both the OOC S and the OPPW 2D OOC C, the code size of S and C are, respectively, M=T and $N=\left\lfloor \frac{1}{\Lambda }\left\lfloor \frac{n-1}{\Lambda -1}\right\rfloor \right\rfloor$ by Lemmas 1 and 2. Thus, applying Theorem 1, we obtain that X is an AM-OPPW 2D OOC with parameters (n×T, Λ,1) and the code size $nNM=nT\left\lfloor \frac{1}{\Lambda }\left\lfloor \frac{n-1}{\Lambda -1}\right\rfloor \right\rfloor$. On the one hand, the fact Λ(Λ−1)∣(n−1) implies that $nT\left\lfloor \frac{1}{\Lambda }\left\lfloor \frac{n-1}{\Lambda -1}\right\rfloor \right\rfloor =nT\frac{n-1}{\Lambda (\Lambda -1)}$, i.e., the code size of X is $nT\frac{n-1}{\Lambda (\Lambda -1)}$. On the other hand, it follows from Lemma 2 that

$$\begin{array}{c} \left|X\right|\leq \left\lfloor \frac{n}{\Lambda }\left\lfloor \frac{T(n-1)}{\Lambda -1}\right\rfloor \right\rfloor =nT\frac{n-1}{\Lambda (\Lambda -1)}, \end{array}$$

where the last equality holds for Λ(Λ−1)∣(n−1). Thus, X is optimal with respect to the bound in Lemma 2. This finishes the proof. □

In Table 1, we list some known optimal OOCs with parameters (n,Λ,1) satisfying Λ(Λ−1)|(n−1), where *p* is a prime and *q* is a prime power.

As an application of Corollary 1, in Table 2, we provide some optimal AM-OPPW 2D OOCs by means of the optimal OPPW 2D OOCs in [19] and optimal OOCs in Table 1.

Next, for λ=2, we have the following corollary.

**Corollary** **2.** *Let C be an optimal OPPW 2D OOC with parameters (Λ×T,Λ,2) and S be an optimal OOC with parameters (n,Λ,2). Then, the AM-OPPW 2D OOC X generated by Construction A with parameters (n×T,Λ,2) is optimal if Λ(Λ−1)(Λ−2)|(n−2) or (Λ−2)|(n−2) and $\Lambda \left(\Lambda -1\right)|\frac{\left(n-1)(n-2\right)}{\Lambda -2}$.*

**Proof.**  The proof is similar to that of Corollary 1. □

It was shown in [30] that there exist optimal OOCs with parameters (n,4,2) for n∈{10,26,34,50,74,98}. Note that (4×3)×2|(n−2) or 2|(n−2) and $\left(4\times 3\right)|\frac{\left(n-1)(n-2\right)}{2}$ for n∈{10,26,34,50,74,98}. Associated with the optimal OPPW 2D OOC of parameters (Λ×p,Λ,2) in [11], where *p* is a prime and 2<Λ≤p, the following result can be directly obtained from Corollary 2.

**Corollary** **3.** *Let C be the optimal OPPW 2D OOC with parameters (4×p,4,2) and S be the optimal OOC with parameters (n,4,2), where p≥4 is a prime and n∈{10,26,34,50,74,98}. Then, AM-OPPW 2D OOC generated from Construction A is optimal with parameters (n×p,4,2).*

**Remark** **1.** *Compared with 2D OOCs, the AM-OPPW 2D OOC may have a lower code rate because the AM-OPPW condition is indeed a constraint from the perspective of code construction. The reader may refer to [12] for a more detailed comparison.*

## 4. Performance Analysis of the New Optimal AM-OPPW 2D OOCs

Let X be the optimal AM-OPPW 2D OOC with parameters (n×T,Λ,1) and code size nMN generated from Corollary 1 based on a known AM-OPPW 2D OOC with parameters (Λ×T,Λ,1) and code size *M* together with a known OOC with parameters (n,Λ,1) and code size *N*. From now on, we examine its performances under the chip-synchronous and chip-asynchronous assumptions, respectively.

In an OCDMA using on–off keying (OOK), “1” and “0” are sent with equal probability but only bit “1” is encoded by the 2D OOC. Following the simple protocol in [1], we analyze the performance of the OCMDA in an ideal case where performance deterioration is only due to multiple-access interference (MAI) so that the effects of physical noises, such as thermal noise, shot noise, and beat noise are ignored [31]. That is, a decision error occurs only when the accumulative MAI reaches over a decision threshold and a data bit zero is transmitted. In addition, before correlation is performed, a hard-limiter is often placed at the front end of a receiver for reducing the effects of MAI [32]. Thus, throughout this section we discuss the performances of the new 2D OOC in the ideal case with a hard-limiter.

### 4.1. Performance Analysis under the Chip-Synchronous Assumption

Without loss of generality, let $X^{\left(C^{0},S^{0},0\right)}$ be the desired codeword. Let $q_{l}$ be the probability of *l* hits in a time slot when it cross-correlates with all the other codewords $X^{\left(C^{i_{1}},S^{r_{1}},j_{1}\right)}$, where 0≤l≤1, $0\leq i_{1}<M$, $0\leq r_{1}<N$, $0\leq j_{1}<n$, and (i,r,j)≠(0,0,0).

For the chip-synchronous case, the hard-limiting error probability of the new AM-OPPW 2D OOCs with parameters (n×T,Λ,λ) in on–off keying (OOK) data modulation is [33]

(3) Psyn=12∑j=ΔΛΛj∑i=0j(−1)j−iji∑m=0λmimΛqmK−1,

where *K* denotes the number of simultaneous users and Δ is the decision threshold. Hence, for the case λ=1, to derive the error probability of the new AM-OPPW 2D OOCs we only need to calculate the probabilities $q_{0}$ and $q_{1}$.

Firstly, we count the number of the hits between arrays $X^{\left(C^{0},S^{0},j0\right)}$ and $X^{\left(C^{i_{1}},S^{r_{1}},j_{1}\right)}$ to compute $q_{1}$. Recall from Algorithm 1 that their *k*-th rows $X_{k}^{\left(C^{0},S^{0},0\right)}\neq 0$ and $X_{k}^{\left(C^{i_{1}},S^{r_{1}},j_{1}\right)}\neq 0$ if and only if

(4) $$\begin{array}{c} s_{k}^{0}=s_{k+j_{1}}^{r_{1}}=1,\;\;\;\;0\leq k<n. \end{array}$$

When $s_{k+0}^{0}=s_{k+j_{1}}^{r_{1}}=1$, one hit occurs exactly once as τ running through all the possible time delays, i.e., 0≤τ<T, since the two rows $X_{k}^{\left(C^{0},S^{0},0\right)}$ and $X_{k}^{\left(C^{i_{1}},S^{r_{1}},j_{1}\right)}$ contain exactly one element 1, respectively. Note that the OOCs $S^{0}$ and $S^{r_{1}}$ have weight Λ. According to the proof of Theorem 1, the following are true:

- When $r_{1}\neq 0$, (4) happens exactly $\Lambda^{2}$ times as $j_{1}$ ranges from 0 to n−1. Then, there are $M\left(N-1\right)\Lambda^{2}$ hits since $i_{1}$ and $r_{1}$, respectively, have *M* and N−1 possible choices;
- When $r_{1}=0$ and $j_{1}\neq 0$, (4) happens exactly $\Lambda^{2}-\Lambda$ times as $j_{1}$ ranges from 1 to n−1. Then, there are $M(\Lambda^{2}-\Lambda )$ hits since $i_{1}$ has *M* possible choices;
- When $r_{1}=0$ and $j_{1}=0$, (4) happens exactly Λ times. Then, there are (M−1)Λ hits since $i_{1}$ has M−1 possible choices.

Therefore, there are $\Lambda^{2}NM-\Lambda$ hits in total. Then, we have

(5) $$q_{1}=\frac{\Lambda^{2}NM-\Lambda }{2T(nMN-1)},$$

where the factor 1/2 comes from the assumptions that the error occurs only if a data bit zero is transmitted and the element 0 is sent with probability 1/2. (nMN−1) denotes the number of codewords except for $X^{\left(C^{0},S^{0},0\right)}$, and *T* is the number of all the time slots. Secondly, the fact $q_{0}+q_{1}=1$ implies

(6) $$q_{0}=1-\frac{\Lambda^{2}NM-\Lambda }{2T(nMN-1)}.$$

In the sequel, we present some simulation results acquired by MATLAB r2023b as an example.

**Figure 1:**
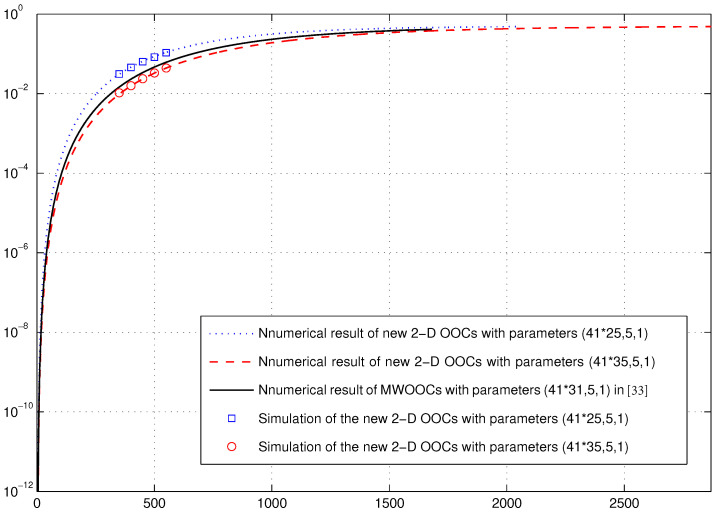
Error probabilities versus the numbers of simultaneous users under the chip-synchronous assumptions [33].

**Example** **1.** *Let C be the optimal OPPW 2D OOC with parameters (5×25,5,1) and code size M=25 [19] and S be the optimal OOC with parameters (41,5,1) and code size N=2 [5]. Then, we can construct a new optimal AM-OPPW 2D OOC with parameters (41×25,5,1) and code size nMN=41×25×2=2050 using Construction A. In this case, using (5) and (6) we obtain $q_{1}=0.0122$ and $q_{0}=0.9878$. Then, we can calculate the hard-limited chip-synchronous error probability by means of (3), which is plotted against K simultaneous users with threshold Δ=Λ=5 in Figure 1. Similarly, based on the OPPW 2D OOC with parameters (5×35,5,1) [19], an optimal AM-OPPW 2D OOC with parameters (41×35, 5,1) and code size nMN=41×35×2=2870 can also be yielded by Construction A.*

*In [34], the multi-wavelength optical orthogonal codes (MWOOCs) with parameters (41×31, 5,1) and code size 1681 can be generated. As a comparison, we plot the hard-limited chip-synchronous error probability of the MWOOCs with parameters (41×31, 5,1) and our new 2D OOCs with parameters (41×25,5,1) and (41×35,5,1) together in Figure 1.*

*Further, simulation for the new 2D OOCs with parameters (41×25,5,1) ((41×35,5,1) resp.) is performed by choosing K codewords for K simultaneous users randomly from the 2050 (2870 resp.) codewords. Particularly, the transmission time delay of each codeword is chosen from a random integer in [0,25) to simulate the chip-synchronous condition. In order to attain the error probability, the simulation is iterated $10^{4}$ times for K∈{350,400,450,500,550}.*

*As shown in Figure 1, the new 2D OOC with parameters (41×35,5,1) has better performance than the MWOOC with parameters (41×31,5,1), while the MWOOC performs better than the new 2D OOC parameters (41×25,5,1). However, the code size of the new 2D OOCs, even for the case with parameters (41×25,5,1), are larger than that of the MWOOCs. This is desirable. On the one hand, the larger the code size, the more the users in the OCDMA systems. On the other hand, new 2D OOCs with large size may support multicode keying in OCDMA systems [35].*

### 4.2. Performance Analysis under the Chip-Asynchronous Assumption

It is known that the chip-synchronous assumption provides pessimistic upper bounds on the performance of the system, whereas the chip-asynchronous assumption assures a more accurate performance [32]. In this subsection, we study the hard-limiting performance of the new AM-OPPW 2D OOCs under the chip-asynchronous assumption.

For the chip-asynchronous case, the hard-limiting error probability of the new AM-OPPW 2D OOCs with parameters (n×T,Λ,λ) in on–off keying (OOK) data modulation is [17,33]

Pasyn=12∑r=ΔΛΛr∑j=0Λ−rΛ−rj2j·∑i=02r+j(−1)2r+j−i2r+ji·∑k=0λ∑l=0λqk,lik+l2Λk+lK−1,

where *K* denotes the number of simultaneous users, $q_{i,j}$ denotes the probability of the cross-correlation value in the preceding time slot equal to 0≤i≤λ (the present time slot 1≤j≤λ, respectively), and Δ is the decision threshold. In particular, for the new AM-OPPW 2D OOCs with parameters (n×T,Λ,1), we then have

(7) Pasyn=12∑r=ΔΛΛr∑j=0Λ−rΛ−rj2j·∑i=02r+j(−1)2r+j−i2r+ji·q0,0+(q0,1+q1,0)i2w+q1,1i22Λ2K−1.

That is, it is sufficient to determine $q_{i,j}$, i,j∈{0,1} for computing $P_{asyn}$.

According to [36], the 2D OOCs with λ=1 satisfy that

(8) $$q_{1,0}=q_{0,1},$$

(9) $$q_{1,1}=q_{1}-q_{0,1},$$

(10) $$q_{0,0}=1-q_{1,1}-q_{1,0}-q_{0,1}.$$

To derive $q_{1,1}$, we need to count the total number of two consecutive hits, i.e., two hits occurring firstly at the preceding time slot and subsequently the present time slot, when the desired code array correlates with all the other arrays in the code set. Without loss of generality, assume that $X^{\left(C^{0},S^{0},0\right)}$ is the desired array.

Firstly, we discuss the arrays from the set $\left\{X^{\left(C,S,j\right)}\;|\;C\in C\;\;\mathrm{and}\;\;\left(S\neq S^{0}\;\;\mathrm{or}\;\;j\neq 0\right)\right\}$. Assume that there exists a hit at the time slot τ, i.e., $R_{X^{\left(C^{0},S^{0},0\right)},X^{\left(C,S,j\right)}}\left(\tau \right)=1$. Note that for $S\neq S^{0}$ or j≠0, there exists at most one integer *k*, 0≤k<n, such that $s_{k+j}=s_{k}^{0}=1$ since their non-trivial correlation value is no more than 1. Then, by Algorithm 1, for any C∈C and any 0≤j≤n−1, both $X_{k}^{\left(C,S,j\right)}\neq 0$ and $X_{k}^{\left(C^{0},S^{0},0\right)}\neq 0$ occur at most once for all the integers 0≤k<n, which indicates that $X^{\left(C,S,j\right)}$ and $X^{\left(C^{0},S^{0},0\right)}$ have at most one hit for all the time slots. This is to say, no other hits happen except for the one at time slot τ.

Secondly, we investigate the arrays based on the same OOC $S^{0}$ and j=0, i.e., $\left\{X^{\left(C,S^{0},0\right)}\;|\;C\in C\right\}$. Suppose that there is a hit at the time slot τ, i.e., $R_{X^{\left(C^{0},S^{0},0\right)},X^{\left(C,S^{0},0\right)}}\left(\tau \right)=1$. In the OOC $S^{0}$, there are Λ elements $s_{k}^{0}=1$ where 0≤k<n. If $s_{k}^{0}=1$, the rows $X_{k}^{\left(C,S^{0},0\right)}$ and $X_{k}^{\left(C^{0},S^{0},0\right)}$ contain exactly one element 1 otherwise they are all-zero vectors according to Algorithm 1. Then, there are Λ possible time slots τ such that $R_{X^{\left(C^{0},S^{0},0\right)},X^{\left(C,S^{0},0\right)}}\left(\tau \right)=1$ and Λ−1 times $\tau^{\prime }$ such that $R_{X^{\left(C^{0},S^{0},0\right)},X^{\left(C,S^{0},0\right)}}\left(\tau^{\prime }\right)=1$ when $\tau^{\prime }$ varies over {0,1,…,T−1}∖{τ} for a given τ. Thus, in total, Λ(Λ−1)(M−1) hits happen as *C* ranging over the set $C\setminus \{C^{0}\}$. This to say, there are $\frac{\Lambda (\Lambda -1)(M-1)}{T-1}$ consecutive hits on average.

Based on the above analysis, we have

(11) $$q_{1,1}=\frac{\Lambda (\Lambda -1)(M-1)}{2T(nMN-1)(T-1)},$$

where the factor 1/2 comes from the assumptions that the error occurs only if a data bit zero is transmitted and the element 0 is sent with probability 1/2, (nMN−1) is the number of codewords except $X^{\left(C^{0},S^{0},0\right)}$, and *T* is the number of all the time slots.

Then, by (8)–(10), we get

(12) $$\begin{array}{c} q_{1,0}=q_{0,1}=q_{1}-q_{1,1}=\frac{\Lambda^{2}NM-\Lambda }{2T(nMN-1)}-\frac{\Lambda (\Lambda -1)(M-1)}{2T(nMN-1)(T-1)}, \end{array}$$

and

(13) $$\begin{array}{c} q_{0,0}=1-q_{1,1}-q_{1,0}-q_{0,1}=1-\frac{\Lambda^{2}NM-\Lambda }{T(nMN-1)}+\frac{\Lambda (\Lambda -1)(M-1)}{2T(nMN-1)(T-1)}. \end{array}$$

Finally, we present some simulation results obtained using MATLAB as an example.

**Figure 2:**
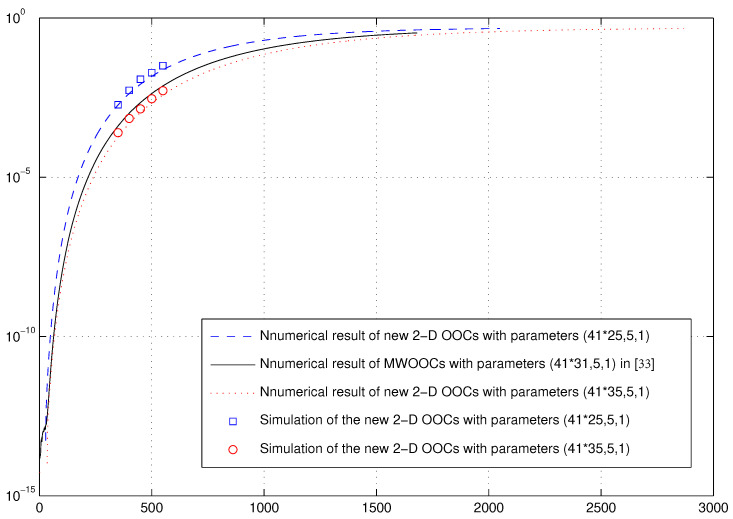
Error probabilities versus the numbers of simultaneous users under chip-asynchronous assumptions [33].

**Example** **2.** *Analyzing the new 2D OOCs with parameters (41×25,5,1) and (41×35,5,1) generated in Example 1, using *(11)*, *(13)*, and *(12)*, we can calculate that $q_{11}=0.0002$, $q_{00}=0.9759$ and $q_{10}=0.0120$ ($q_{11}=0.0001$, $q_{00}=0.9827$ and $q_{10}=0.0086$ resp.). Substituting them into (7), we derive the hard-limited chip-asynchronous error probability, which is plotted against K simultaneous users in Figure 2, where Δ=Λ=5. As a comparison, we also plot the hard-limited chip-asynchronous error probability of the MWOOCs with parameters (41×31,5,1) together with our new 2D OOCs with parameters (41×25,5,1) and (41×35,5,1) in Figure 2.*

*Simulations are conducted by choosing K codewords for K simultaneous users randomly from 2050 (2870 resp.) codewords. Specifically, the transmission time of each codeword is chosen from a random real number between 0 and 25 to simulate the chip asynchronous condition. In order to acquire an error probability, the simulations are iterated $10^{4}$ times for K∈{350,400,450,500,550}. As shown in Figure 2, the simulation result is very close to the chip-asynchronous curves given by* (7).

**Figure 3:**
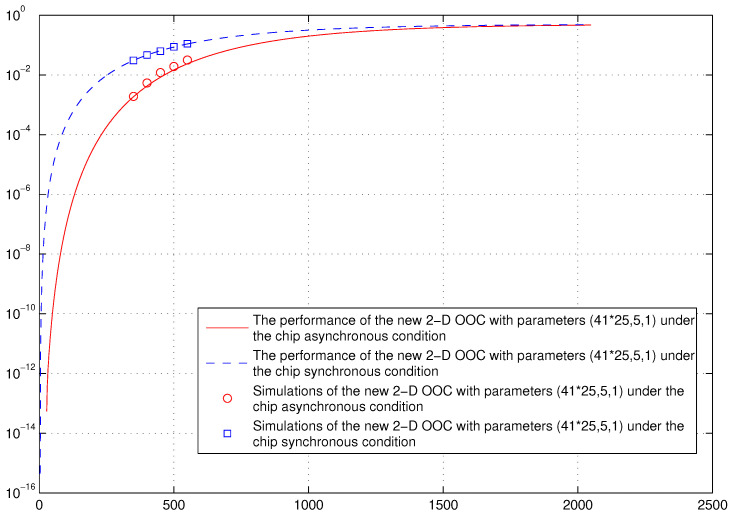
Error probabilities of the new optimal AM-OPPW 2D OOC under hard-limited chip-asynchronous and hard-limited chip synchronous conditions.

*In addition, we compare the performance of the new optimal AM-OPPW 2D OOC with parameters (41×25,5,1) under the hard-limited chip asynchronous and hard-limited chip synchronous conditions in Figure 3. It is seen that the performance of the hard-limited chip asynchronous is better than the hard-limited chip synchronous case, which is consistent with the result in [32].*

**Figure 4:**
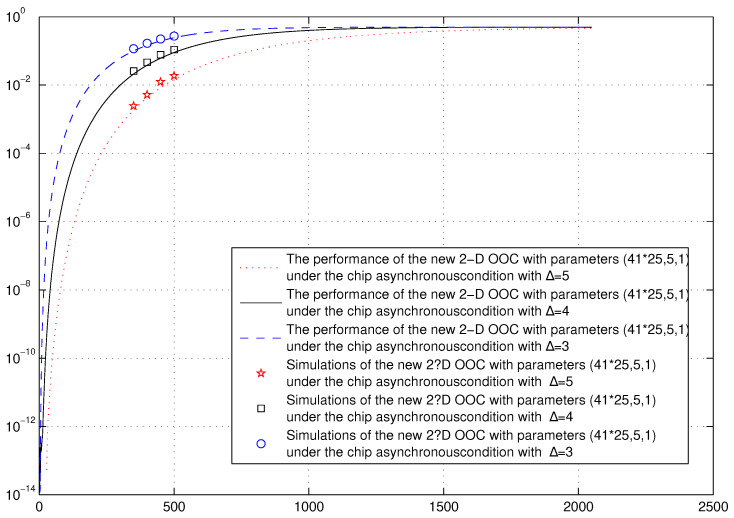
Error probabilities of the new optimal AM-OPPW 2D OOC under the hard-limited chip asynchronous with different decision threshold Δ.

*In Figure 4, we plot the performance of the new 2D OOC with parameters (41×25,5,1) under hard-limited chip-asynchronous conditions for the decision threshold Δ with the value varying from 3 to 5. It was firstly pointed out in [32] that the higher the threshold level, the better system performance since multiple users will become less probable to occupy a particular chip above the level of the threshold. Clearly, our simulation result reveals this fact.*

## 5. Conclusions

In this paper, a new generic construction of AM-OPPW 2D OOCs was proposed. By restricting the OOCs and OPPW 2D OOCs to optimal ones, optimal AM-OPPW 2D OOCs and asymptotically optimal 2D OOCs with new parameters were obtained. Additionally, the performance of the new AM-OPPW 2D OOCs was demonstrated under both chip-synchronous and chip-asynchronous assumptions.

However, in general, the known parameters of AM-OPPW 2D OOCs are quite limited, and the performance of AM-OPPW 2D OOCs in real-world scenarios remains an open question.

## Figures and Tables

**Table 1:** Some known optimal OOCs with Λ(Λ−1)|(n−1).

Parameters	Code Size	Constraint	Ref.
$\left(q^{2}+q+1,q+1,1\right)$	1		[4,28]
$\left(\frac{q^{d+1}-1}{q-1},q+1,1\right)$	$\frac{q^{d}-1}{q^{2}-1}$	*d* even	[4]
(n,3,1)	$\frac{n-1}{6}$	n≡1(mod6)	[4]
(p,w,1)	*r*	p=w(w−1)r+1	[29]

**Table 2:** Some new optimal AM-OPPW 2D OOCs from Corollary 1.

Parameters	Code Size	Constraint
$\left(\left(q^{2}+q+1\right)\times T,q+1,1\right)$	$(q^{2}+q+1)T$	$T=p_{k}p_{k-1}\ldots p_{1}$
		with $p_{k}\geq p_{k-1}\geq p_{1}\geq q+1$
$\left(\frac{q^{d+1}-1}{q-1}\times T,q+1,1\right)$	$\frac{T\left(q^{d+1}-1\right)\left(q^{d}-1\right)}{\left(q-1\right)\left(q^{2}-1\right)}$	$T=p_{k}p_{k-1}\ldots p_{1}$
		with $p_{k}\geq p_{k-1}\geq p_{1}\geq q+1$
(n×T,3,1)	$\frac{Tn(n-1)}{6}$	$T=p_{k}p_{k-1}\ldots p_{1}$
		with $p_{k}\geq p_{k-1}\geq p_{1}\geq 3$
		n≡1mod6
(p×T,w,1)	rpT	p=w(w−1)r+1
		$T=p_{k}p_{k-1}\ldots p_{1}$
		with $p_{k}\geq p_{k-1}\geq p_{1}\geq w$

## Data Availability

The original contributions presented in the study are included in the article, further inquiries can be directed to the corresponding author.
